# Degradation of edible mushroom waste by *Hermetia illucens* L. and consequent adaptation of its gut microbiota

**DOI:** 10.1038/s41598-024-60524-6

**Published:** 2024-04-30

**Authors:** Linhui Lai, Yaohang Long, Meng Luo, Bo Tu, Zailin Wu, Jinling Liu, Zhixian Wan, Guangyin Wang, Xianyi Wang, Hongmei Liu

**Affiliations:** 1 School of Public Health, the key Laboratory of Environmental Pollution Monitoring and Disease Control, Ministry of Education, Guizhou Medical University, Guiyang, 561113 China; 2 Engineering Research Center of Medical Biotechnology, School of Biology and Engineering, Guizhou Medical University, Guiyang, China; 3 Engineering Research Center of Health Medicine Biotechnology of Institution of Higher Education of Guizhou Province, Guiyang, China; 4 Key Laboratory of Biology and Medical Engineering, Immune Cells and Antibody Engineering Research Center of Guizhou Province, School of Biology and Engineering, Guizhou Medical University, Guiyang, China; 5 School of Basic Medicine Science, Guizhou Medical University, Guiyang, China

**Keywords:** *Hermetia illucens*, Edible fungi, Mushroom waste, Intestinal microenvironment, Ecology, Agroecology

## Abstract

The edible fungus industry is one of the pillar industries in the Yunnan–Guizhou Plateau, China. The expansion of the planting scale has led to the release of various mushroom residues, such as mushroom feet, and other wastes, which are not treated adequately, resulting in environmental pollution. This study investigated the ability of black soldier fly (*Hermetia illucens L*.) larvae (BSFL) to degrade mushroom waste. Moreover, this study analyzed changes in the intestinal bacterial community and gene expression of BSFL after feeding on mushroom waste. Under identical feeding conditions, the remaining amount of mushroom waste in *Pleurotus ostreatus* treatment group was reduced by 18.66%, whereas that in *Flammulina velutipes* treatment group was increased by 31.08%. Regarding gut microbial diversity, compared with wheat bran-treated control group, *Dysgonomonas*, *Providencia*, *Enterococcus*, *Pseudochrobactrum*, *Actinomyces*, *Morganella*, *Ochrobactrum*, *Raoultella*, and *Ignatzschineria* were the most abundant bacteria in the midgut of BSFL in *F. velutipes* treatment group. Furthermore, *Dysgonomonas*, *Campylobacter*, *Providencia*, *Ignatzschineria*, *Actinomyces*, *Enterococcus*, *Morganella*, *Raoultella*, and *Pseudochrobactrum* were the most abundant bacteria in the midgut of BSFL in *P. ostreatus* treatment group. Compared with wheat bran-treated control group, 501 upregulated and 285 downregulated genes were identified in *F. velutipes* treatment group, whereas 211 upregulated and 43 downregulated genes were identified in *P. ostreatus* treatment group. Using Kyoto Encyclopedia of Genes and Genomes and Gene Ontology enrichment analyses, we identified 14 differentially expressed genes (DEGs) related to amino sugar and nucleotide sugar metabolism in *F. velutipes* treatment group, followed by 12 DEGs related to protein digestion and absorption. Moreover, in *P. ostreatus* treatment group, two DEGs were detected for fructose and mannose metabolism, and two were noted for fatty acid metabolism. These results indicate that feeding on edible mushroom waste can alter the intestinal microbial community structure of BSFL; moreover, the larval intestine can generate a corresponding feedback. These changes contribute to the degradation of edible mushroom waste by BSFL and provide a reference for treating edible mushroom waste using BSFL.

## Introduction

During the process of cultivation, edible fungi produce waste products such as fungus bran and mushroom waste, which lead to huge amounts of waste and ultimately environmental pollution. Currently, treatment of fungus bran is a relatively mature technology, whereas the rational use of mushroom waste remains relatively insufficient. The term “edible mushroom waste” includes the waste corresponding to edible fungus roots and the small amount of fungus bran present at the base of edible fungus stalks (1–3 cm)^1^. During the cultivation of *Flammulina velutipes* alone, China produces 100,000 tons of dry waste annually^2^. In addition to dietary fiber, edible mushroom waste contain proteins, polysaccharides, and other important nutrients. Currently, mushroom waste are mainly treated via incineration or landfill. Occasionally, they are also used as fertilizers; however, their overall utilization level is low^3^. The use of resource-based invertebrates or insects is a new approach that has recently been reported for treating edible mushroom waste. A previous study explored the manner in which earthworms recycle organic waste from the soil of edible fungi and transform it into compost for crop cultivation^4^. Another study reported the use of edible mushroom residue to feed gurb and achieve waste recycling^5^. The black soldier fly *Hermetia illucens* L. (Diptera: *Stratiomyidae*), an omnivorous saprophytic insect that can degrade mushroom waste, is an important resource insect; however, its specific degradation ability remains unclear.

The black soldier fly has a short life cycle and is easy to breed and grow^6,7^. Black soldier fly larvae (BSFL) can convert organic waste such as food waste^8^ into larval biomass that is rich in proteins and lipids^8–10^, which can be used as feed addition in poultry and fishery industries. The resulting insect sand is also an excellent nitrogen-rich organic fertilizer^11–13^, which has attracted worldwide attention, resulting in the large-scale breeding of this insect^14,15^. Concomitantly, the BSFL feeding activities have significantly reduced a large amount of organic waste and environmental pollutants^16,17^. The digestion and degradation abilities of BSFL are mainly attributable to larval intestinal and intestinal microorganisms^18^. In the gut of BSFL, enzyme-producing bacteria participate in the conversion and utilization of nutrients by the host; moreover, bacteria possessing the ability to decompose organophosphorus have been isolated from the gut of BSFL^19^, and microbiota with digestive enzyme activities, such as protease^20^, lipase^21^, and amylase^22^ activities, have been detected in the midgut of the insect. Previous studies have shown that the feeding medium of insects can affect their growth rate and nutrient content^23,24^. Furthermore, a study revealed that different dietary structures can alter the intestinal bacterial composition of BSFL, thus ensuring that it can adapt to dietary changes, meet the nutritional intake of different foods, and increase the adaptability of BSFL to the environment^25^.

This study aimed to explore the ability of BSFL to degrade mushroom waste, which are easy to digest for humans and difficult to digest as research objects. In this study, *Pleurotus ostreatus*, which is easy to digest for humans, and *F. velutipes*, which is difficult to digest, were selected as the research objects to explore the ability of BSFL to degrade mushroom waste. BSFL fed with the mushroom waste of *F. velutipes* and *Pleurotus ostreatus* were compared with wheat bran-treated control group to calculate the forage of each BSFL group after feeding on mushroom waste. Intestinal microbial diversity analysis and intestinal transcriptome sequencing technology were used to explore changes in intestinal microbial community structure and function in different treatment groups at the microbial community level. Moreover, this study explored changes in intestinal gene expression at the transcriptome level. Our results provide a new reference for the degradation of mushroom waste and propose an approach for reducing environmental pollution caused by agricultural waste.

## Materials and methods

### Source of the tested insect and mushrooms

The BSFL used in this study were obtained from a colony reared at the Industrial Research Group, College of Biology and Engineering. The BSFL were reared under controlled environmental conditions (T = 28 °C ± 1 °C, relative humidity [RH] = 70% ± 10%) until the 5th-instar larval stage and were used as the insect source in this study. The mushroom waste used in this study were obtained from the canteen of Guizhou Medical University and were selected as the experimental material.

### Treatment of mushroom waste with 5th-instar BSFL

The experimental group was divided into nine parallel groups, as follows: three groups each for treatment with *F. velutipes*, *P. ostreatus*, and wheat bran (which was used as the control). Each parallel group was weighed to ensure that it contained 105 g of BSFL with a similar body size. The BSFL used weighs about 0.105 g each and has a body length of 12–19 mm. Subsequently, the three parallel groups were incubated with 750 g of *F. velutipes* mushroom waste, three parallel groups were incubated with 750 g of *P. ostreatus* mushroom waste, and the remaining three parallel groups were incubated with 750 g of wheat bran. Nine additional parallel groups were set up that contained only equal amounts of mushroom waste and wheat bran, without BSFL, as a control group, and were incubated at a feeding temperature of 25–28 °C and humidity of 70–75%. After 5 days of treatment, the amount of remaining edible mushroom waste and quality of wheat bran were recorded. BSFL was also raised in the same way from the 4th-instar stage, and after 12 days of treatment, the intestine and intestinal contents were separated for total DNA extraction, intestinal bacterial library construction, and sequencing.

We collected 5th-instar BSFL with consistent size and health status, rinsed them with sterile water for 30 s, disinfected the body surface with 75% alcohol, and finally washed away the residual alcohol from the body surface using sterile water. Under aseptic conditions, we sliced open the body surface of the larvae from the side, carefully removed the intact intestinal tract, and cut off the midgut. We removed the intestinal contents and collected them in 2-mL sterile centrifuge tubes. The intestinal contents of 30 BSFL were placed in each collection tube, and 3 tubes were prepared for each treatment group. We used PowerSoil® DNA Isolation kit to extract DNA. After quality control, we used Solexa PCR library preparation kit to construct a gene library and sequenced the gene library using the PacBio Sequel sequencing platform.

### Operational taxonomic unit (OTU) clustering, diversity analysis, and functional analysis of intestinal bacterial communities

Usearch software was used to cluster the sequencing reads at a similarity level of 97.0% and obtain the OTU sequence. Using SILVA as a reference database, a naïve Bayesian classifier combined with alignment methods was employed to perform taxonomic annotation of the characteristic sequences. QIIME software was used to generate species abundance tables at different taxonomic levels, and R language tools were employed to construct community structure diagrams at each taxonomic level of the samples. Moreover, QIIME2 software was employed to evaluate the α-diversity index of the samples, including bacterial abundance indexes (Chao and Ace) and bacterial diversity indexes (Shannon and Simpson) for each sample. Subsequently, corresponding dilution curves were constructed to determine whether the sequencing yield was adequate to cover all taxa. Using QIIME software for β-diversity analysis, the similarity of species diversity was compared among different samples via principal component analysis and principal coordinate analysis (PCoA), NMDS, UPGMA, sample clustering heatmap, and Anosim analyses. After the completion of OTU sequence species annotation, the results were directly or indirectly mapped to the related Kyoto Encyclopedia of Genes and Genomes (KEGG)-annotated microbial genomes. The results were further combined with the annotated bacterial abundance of the samples, 16S rRNA gene copy number in the genomes, and functional genes (using KEGG Ortholog, KO representation) to obtain functional gene data and abundance of several known bacteria in the samples. Furthermore, the diversity and metabolic pathways of intestinal bacteria were analyzed in the samples fed with edible mushroom waste and wheat bran, along with analysis of the enzymes involved in the consumption of edible mushroom waste.

### Isolation and identification of extracellular enzyme-producing bacteria in the midgut of BSFL

The midgut of BSFL was collected and rinsed with sterile water for 30 s, and phosphate-buffered saline was added for sample grinding. The grinding liquid suspensions was spread on a plate, and the purification process was repeated via streaking until a single colony appeared on the plate. Next, a single colony from LB liquid medium was picked up and incubated at 37 °C until the bacterial solution became cloudy. Subsequently, 10 μL of the bacterial solution was steadily dropped onto a pre-prepared enzyme activity screening plate (the center of the cellulase enzyme screening plate; protease-, urease-, and asparaginase-detection plate), which was placed in a 37 °C incubator for incubation and observation. After sequencing the isolated strains, NCBI sequence alignment was performed, and a phylogenetic tree was constructed for strain identification.

### Transcriptome RNA extraction, library construction, and sequencing

Total RNA was extracted from the midgut of each treatment group of BSFL using R6834 Total RNA Kit I. Three replicates were used for each treatment group, with each replicate comprising 15 midgut samples of BSFL. After quality control, VAHTS Universal V6 RNA-seq Library Prep Kit (Illumina®, NR604-01/02) was used to select different index tags for library construction. The qualified libraries were sequenced using the HiSeq sequencing platform.

### Data quality control and comparison with the reference genome

Quality control of the filtered data was performed using fastac^26^. The filtered transcriptome sequences were aligned with reference genes according to Star^27^. Finally, statistical analysis was performed.

### Differentially expressed genes (DEGs) and their functional enrichment analysis

The expression level of genes was estimated by counting the sequences located in the genomic region or gene exon region, and functional annotation of transcripts was performed using seven databases, including Nr^28^, Pfam^29^, Uniprot, KEGG^30^, Gene Ontology (GO)^31^, KOG/COG^32^, and PATHWAY^30^. The number of reads that were mapped to each transcript in all samples was obtained using RSEM^33^, and FPKM conversion was performed. Double-ended sequences from the same fragment were counted as one fragment, and the expression levels of genes and transcripts were determined. The quantitative expression levels of the genes in each sample were used for differential expression analysis using DESeq2 software^34^. The screening threshold was *P*adj < 0.05 and |log2FoldChange|> 1, and the main metabolic pathways or signaling pathways associated with the DEGs were determined.

### Ethics approval

This article does not contain any studies with human participants performed by any of the authors.

## Results

### Degradation of edible mushroom waste and wheat bran by 5th-instar BSFL

**Figure 1:**
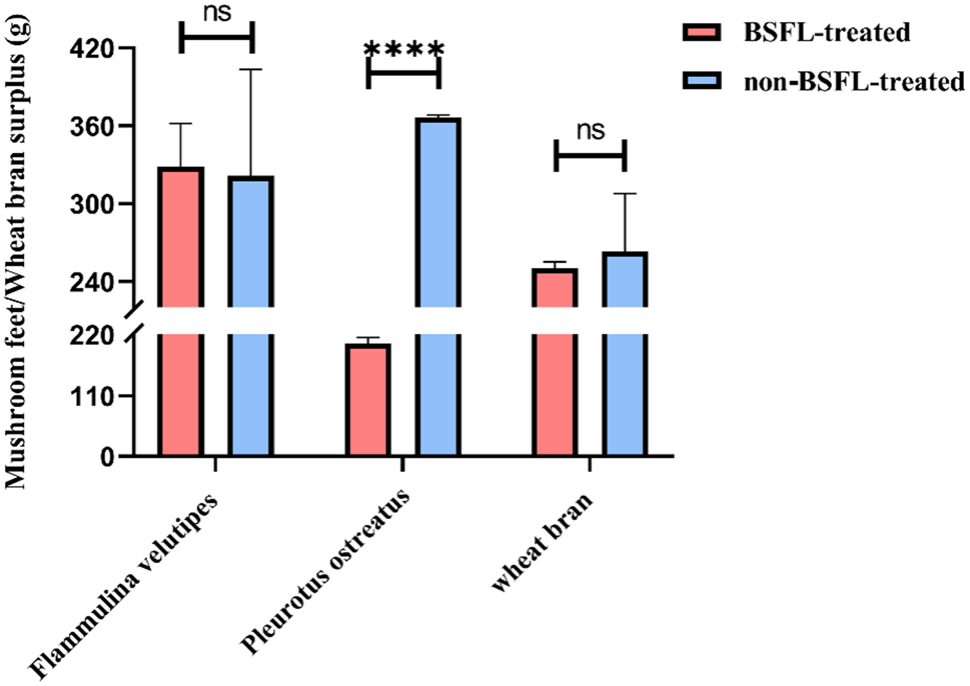
Mushroom waste/wheat bran surplus.

After treatment with BSFL for 5 days, differences were observed in the consumption of *P. ostreatus* waste, *F. velutipes* waste, and wheat bran (Fig. 1). The consumption was compared between BSFL-treated (red) and non-BSFL-treated (blue) groups to determine whether BSFL treatment resulted in statistically significant differences in feed surplus. The remaining amount of BSFL-treated *F. velutipes* waste was 328.3 g, whereas the consumption in the non-BSFL-treated group was 321.733 g, with no statistically significant differences between the two groups. The remaining amount of *P. ostreatus* waste in the BSFL-treated group was 203.733 g, whereas that in the non-BSFL-treated group was 366.533 g, with a statistically significant difference between the two groups. As a control, the amount of wheat bran that remained after BSFL treatment was 250.467 g, whereas that in the non-BSFL-treated group was 263.367 g, with no statistically significant differences between the two groups. BSFL exhibited varying consumption rates for different edible mushroom waste and wheat bran, with the consumption of *P. ostreatus* waste being greater than that of wheat bran and the consumption of *P. ostreatus* waste after BSFL treatment being significantly increased compared with the untreated group. At the same time, the weight of BSFL in three different treatment groups was statistically analyzed. The average weight of BSFL in the *Flammulina velutipes* treatment group decreased by 36.4 g, the average weight of BSFL in the wheat bran control group increased by 56.5 g, and the average weight of BSFL in *Pleurotus ostreatus* treatment group increased by 79.1 g.

### Splicing and assembly of the 16srDNA sequence of the microorganisms present in the midgut of BSFL

A total of 66,971 reads were obtained from the midgut samples of the 5th-instar larvae of the black soldier flies. After quality control, the available sequence reads were clustered at a similarity level of 97.0% (Table 1). The annotation results based on OTUs showed that 117 OTUs were obtained from the combination of 9 samples, with 7 phyla, 9 classes, 22 orders, 42 families, 78 genera, and 101 species being annotated.

**Table 1:** Sequence analysis of the 16S rDNA of gut bacteria detected in the 5th-instar larvae of *Hermetia illucens L.*

Samples	Nmnber of valid sequences	Number of OTUs	Number of different taxonomic categories					
			Phylum	Class	Order	Family	Genus	Species
Jl	6981	84	6	8	19	37	59	79
J2	8029	81	6	8	17	34	56	72
J3	8032	91	6	8	18	36	64	82
Ml	6985	64	6	8	16	26	43	53
M2	7391	66	6	8	17	26	44	55
M3	8094	66	6	8	17	29	47	58
PI	7833	44	6	8	14	21	27	37
P2	8203	58	6	8	IS	30	44	53
P3	7139	71	7	9	22	33	51	65
Total	68687	117	7	9	22	42	78	101

J1-J3, BSFL midgut in *Flammulina velutipes* treatment group; M1-M3, BSFL midgut in wheat bran-treated control group; P1-P3, BSFL midgut in *Pleurotus ostreatus* treatment group.

### Sequencing depth and α-diversity analysis of the midgut microbial community of BSFL

**Figure 2:**
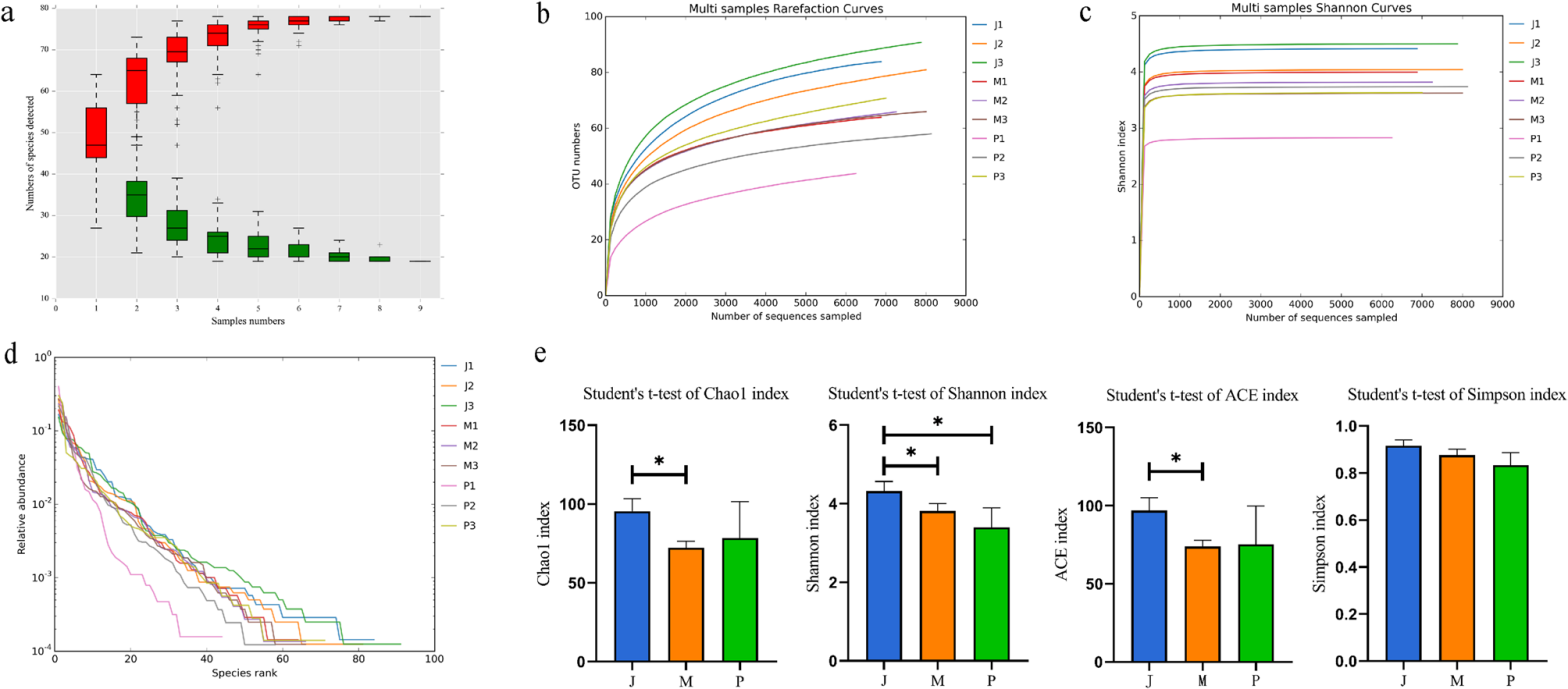
(**a**) Species relative abundance accumulation curve of BSFL gut microbes in each treatment group. (**b**) BSFL gut microbial dilution curve of each treatment group. (**c**) BSFL gut microbial Shannon index curve of each treatment group. (**d**) Grade abundance curves of BSFL intestinal microorganisms in different treatment groups. (**e**) BSFL gut microbial α-diversity of each treatment group, as estimated using Chao1, Shannon, ACE, and Simpson indexes.

The horizontal species accumulation curve (Fig. 2a), rarefaction curve (Fig. 2b), and Shannon diversity index dilution curve (Fig. 2c) tended to be smooth at the end, indicating a sufficient sequencing sample size. The rank abundance curve (Fig. 2d) was wide and flat, indicating the presence of abundant sample species and high uniformity. The α-diversity of BSFL intestinal microorganisms in different treatment groups was analyzed using the Chao index, Shannon index, Ace index, and Simpson index. Figure 2e presents the mean α-diversity index of different treatment groups. As shown in Table 2, the mean Shannon index, Chao index, and Simpson index of BSFL intestinal microorganisms in mushroom treatment group were 4.32, 95.57, and 0.92, respectively. Furthermore, the mean Shannon index, Chao index, and Simpson index of BSFL intestinal microorganisms in wheat bran-treated control group were 3.81, 72.21, and 0.88, respectively. Moreover, in mushroom treatment group, the corresponding mean values were 3.4, 78.33, and 0.83. The species abundance and diversity of BSFL intestinal microorganisms in mushroom treatment group were higher than those in wheat bran-treated control group.

**Table 2:** α-diversity indexes of bacteria in the midgut of the 5th-instar larvae of *Hermetia illucens* L.

Samples	Diversity index		
	Chaol	Shannon	Simpson
J	95.57	4.32	0.92
M	72.21	3.81	0.88
P	78.33	3.4	0.83
Total	7	9	22

### Analysis of bacterial β-diversity in the midgut of BSFL

**Figure 3:**
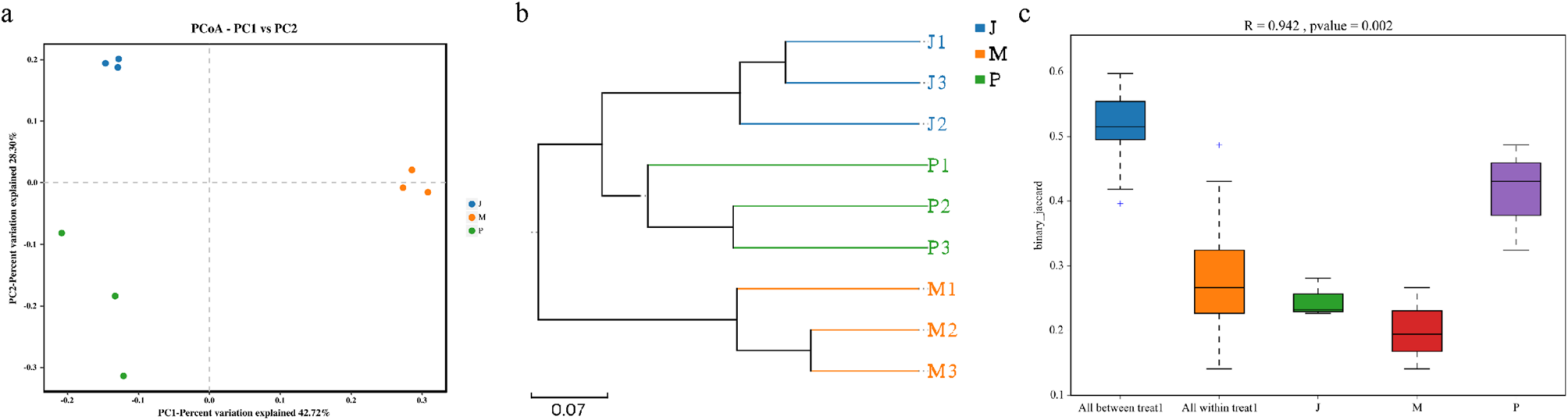
(**a**) PCoA of BSFL gut microbes in each treatment group based on the weighted UniFrac distance matrix. (**b**) UPGMA clustering tree of BSFL gut microbes in each treatment group. (**c**) Anosim analysis box of BSFL gut microbes in each treatment group. As the *R* value became closer to 1, the difference between the groups became greater than the difference within groups, and *P*-values of < 0.05 indicated high reliability of the test. The ordinate represents the β distance. The blue box diagram represents the β distance data of all intergroup samples, the orange box diagram represents the β distance data of all intragroup samples, and the subsequent box diagram represents the β distance data of intragroup samples of different groups.

The results of PCoA (Fig. 3a) showed that there were nine samples from *F. velutipes* treatment group, wheat bran-treated control group, and *P. ostreatus* treatment group, and different treatment samples were clustered in different quadrants without any overlap. UPGMA hierarchical clustering (Fig. 3b) of the samples revealed that the samples within the same treatment group were close to each other and had short branches, whereas those in different treatment groups were far away from each other and had long branches. Anosim analysis (Fig. 3c) revealed that the *R* value was 0.942, which was close to 1, and the difference between the groups was greater than the difference within the groups. The *P*-value was 0.002, which was far below 0.05, with high reliability, indicating significant differences in β-diversity between different treatment groups. Differences between the samples in distinct treatment groups were greater than those observed within the same treatment group.

### Comparison of the species and abundance of the microorganisms identified in the midgut of BSFL in the different groups

**Figure 4:**
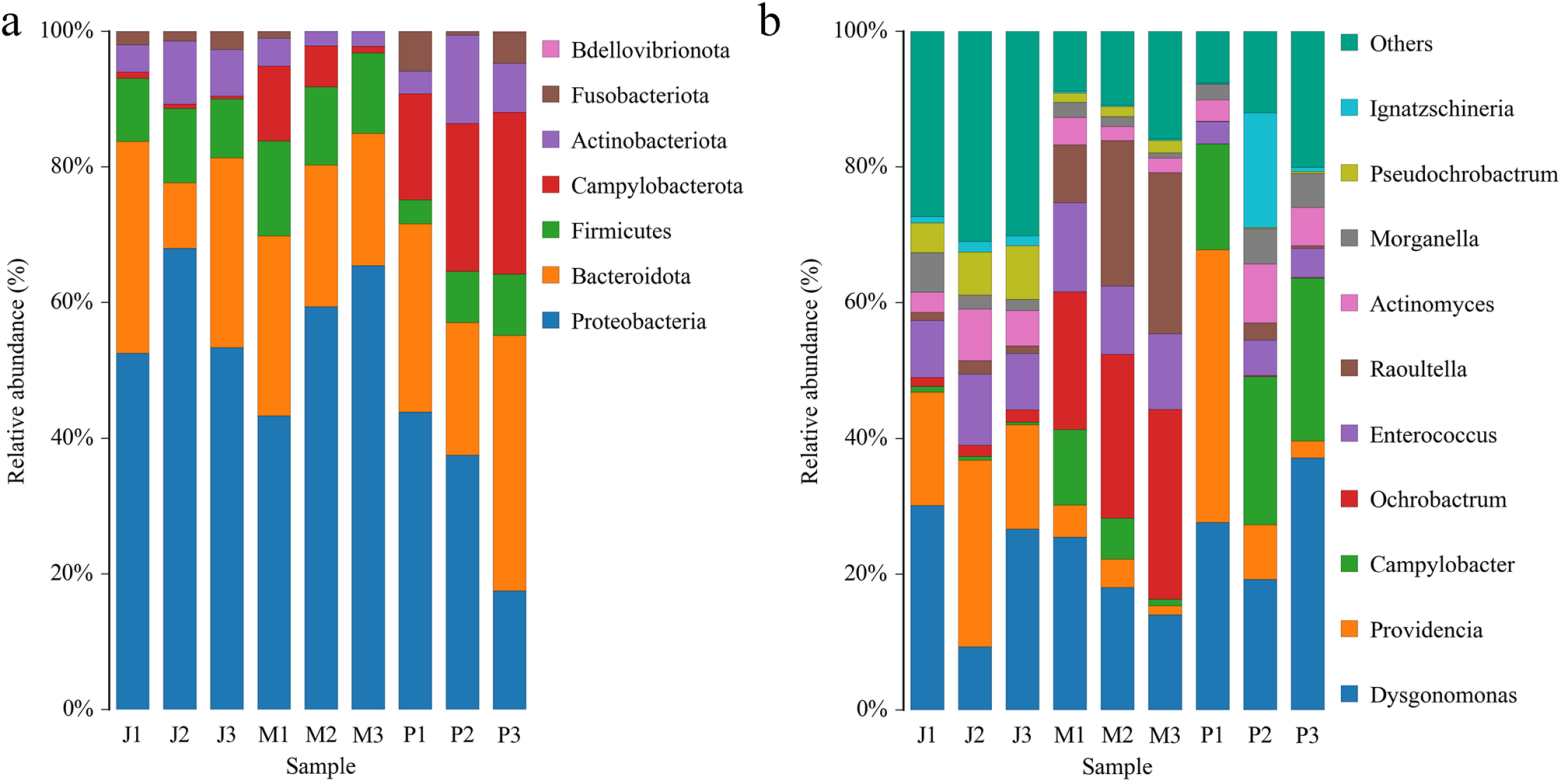
Relative abundance of the dominant phyla (**a**) and genera (**b**) of gut bacteria in the 5th-instar larvae of *Hermetia illucens* L.

Figure 4a shows that the top 3 bacterial species in the BSFL of *F. velutipes* treatment group at the phylum level were Proteobacteria (58.22%), Bacteroidota (22.57%), and Firmicutes (9.68%). The top 3 bacteria in the BSFL of wheat bran-treated control group at the phylum level were Proteobacteria (56.58%), Bacteroidota (22.12%), and Firmicutes (12.43%). The top 3 bacteria in the BSFL of *P. ostreatus* treatment group at the phylum level were Proteobacteria (32.82%), Bacteroidota (27.89%), and Campylobacterota (20.70%). The top 10 bacteria at the genus level were analyzed (Fig. 4b). The top 10 bacterial genera in the BSFL of *F. velutipes* treatment group were “other” (29.58%), *Dysgonomonas* (21.68%), *Providencia* (19.98%), *Enterococcus* (9.04%), *Pseudochrobactrum* (6.29%), *Actinomyces* (5.36%), *Morganella* (3.07%), *Ochrobactrum* (1.62%), *Raoultella* (1.45%), and *Ignatzschineria* (1.34%). Furthermore, the top 10 bacteria with the highest relative abundance in the midgut of BSFL in wheat bran-treated control group were *Ochrobactrum* (24.34%), *Dysgonomonas* (18.94%), *Raoultella* (18.28%), “other” (12.03%), *Enterococcus* (11.37%), *Campylobacter* (5.77%), *Providencia* (3.30%), *Actinomyces* (2.72%), *Pseudochrobactrum* (1.58%), and *Morganella* (1.42%). The top 10 bacteria with the highest relative abundance in the midgut of BSFL in mushroom treatment group were *Dysgonomonas* (27.59%), *Campylobacter* (20.70%), *Providencia* (15.66%), “other” (13.37%), *Ignatzschineria* (6.63%), *Actinomyces* (6.04%), *Enterococcus* (4.34%), *Morganella* (4.33%), *Raoultella* (1.12%), and *Pseudochrobactrum* (0.16%). The relative abundance ranking of the bacterial species in the midgut of BSFL varied in different treatment groups.

### Detection of extracellular enzyme activity and identification of bacterial species in the gut of BSFL

**Figure 5:**
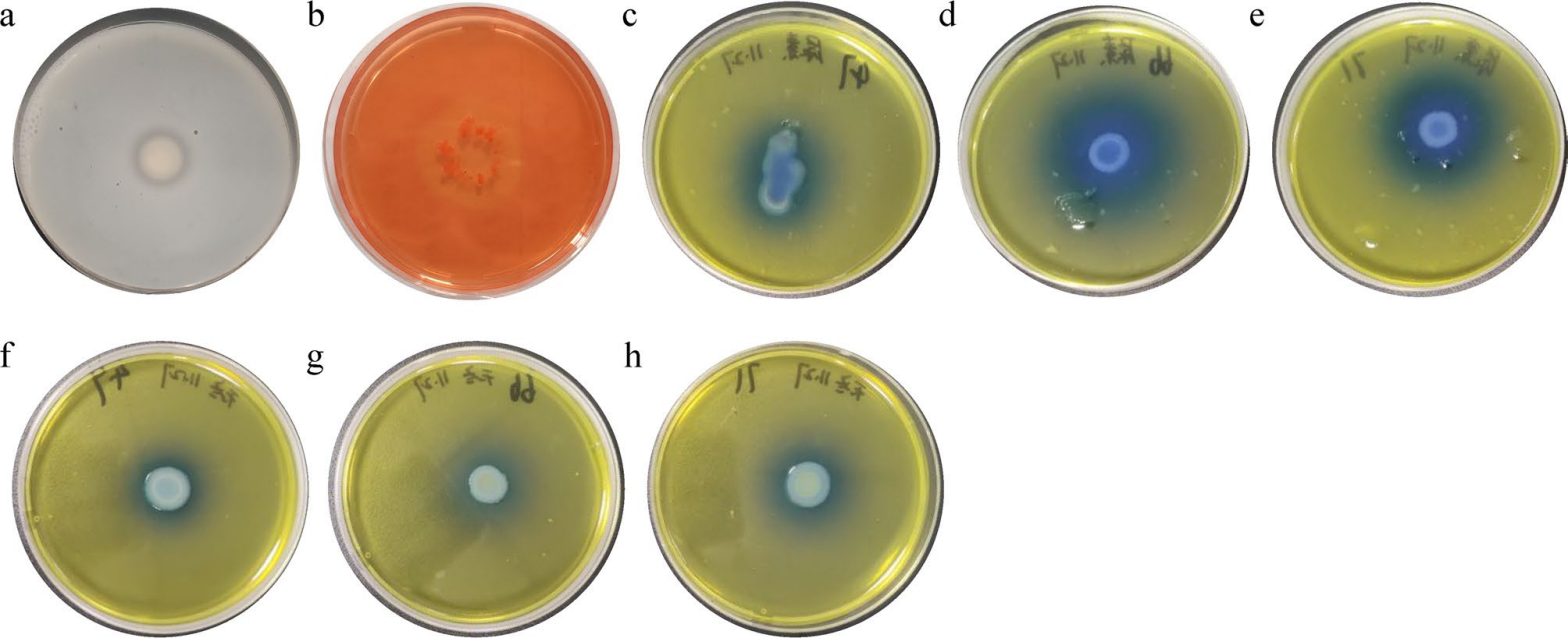
Detection of the extracellular enzyme activity of intestinal bacteria present in the 5th-intar larvae of *Hermetia illucens* L.

Three strains (preliminarily termed 47, 66, and 71) were isolated and purified from the gut of BSFL. After enzyme activity detection on the plate, strain 71 exhibited a clear circle around the cell body on the protease-detection plate (Fig. 5a), indicating the presence of protease activity. Furthermore, strain 47 showed a clear circle around the cell body on the cellulase-detection plate after Congo red staining (Fig. 5b), indicating the presence of cellulase activity. Strains 47, 66, and 71 showed blue areas around the cell body on the urease-detection plate (Fig. 5c–e), indicating that all three strains had urease activity. Strains 47, 66, and 71 showed blue areas around the cell body on the asparaginase-detection plate (Fig. 5f–h), indicating that all three strains had asparaginase activity. Finally, based on sequencing, strains 47, 66, and 71 were identified as *Providencia* sp. (strain 47) and *Proteus mirabilis* (strains 66 and 71).

### Statistical analysis and quality assessment of the gut transcriptome sequencing data of BSFL in each treatment group

After the administration of wheat bran to *F. velutipes* and *P. ostreatus* waste, the total amount of data obtained from the transcriptome sequencing of the BSFL intestinal tract was 630 303 022 sequences, and the sample data amount of each treatment group was 70 004 922-70 050402 sequences. After data filtering and quality control, 100% of the data was retained as clean reads, and 630 303 022 sequences were obtained for downstream analysis, with 70 004 922-70 050402 sequences identified for each treatment group, among which the *F. velutipes* treatment group exhibited the highest number of sequences and the wheat bran-treated control group had the lowest number of sequences. The Q20% of each treatment group was above 97.387%, and the Q30 was above 91.990%. The GC content was 39.733–43.939%. The transcriptome sequencing quality was high, and the data met the requirements for bioinformatics analysis.

### Analysis of DEGs in the gut of BSFL in each treatment group

**Figure 6:**
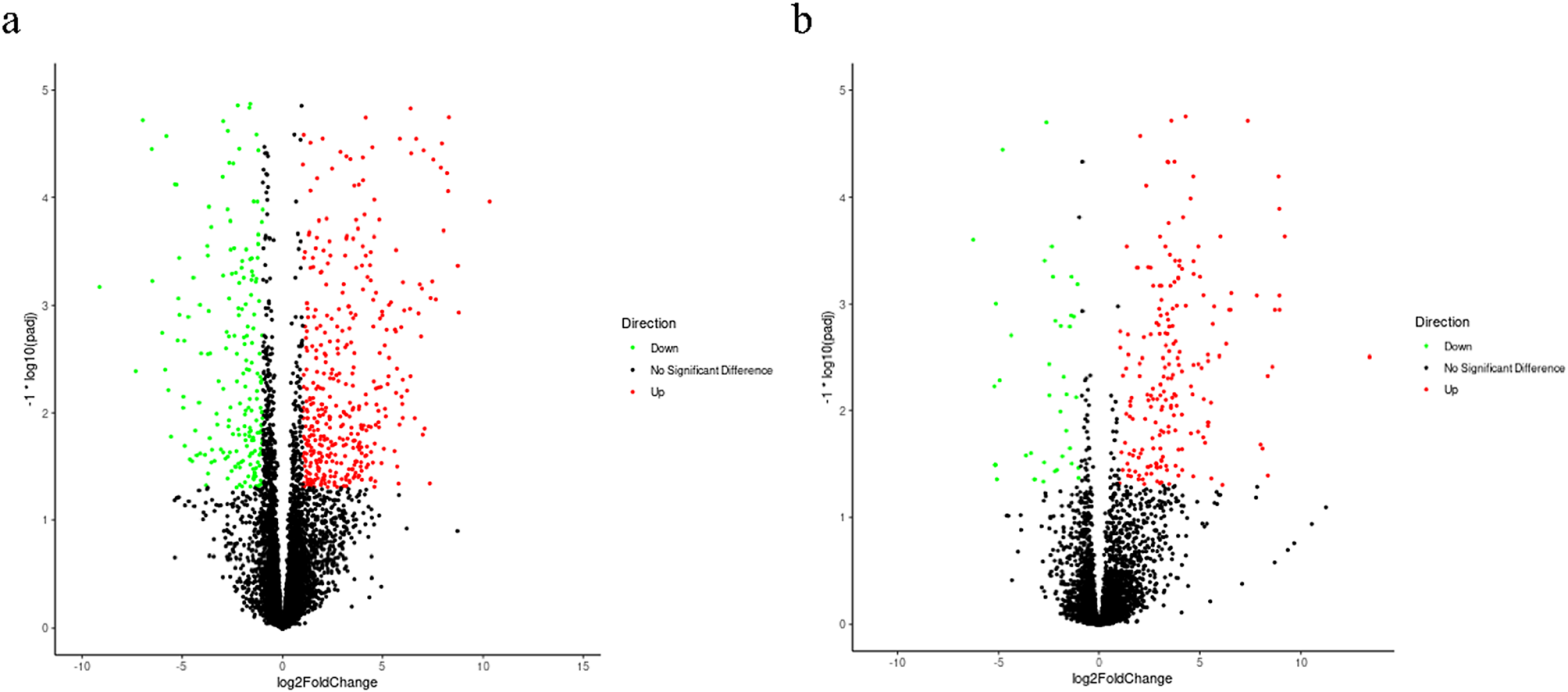
Volcano plot of the gut in the 5th-instar larvae of *Hermetia illucens* L. based on different *Flammulina velutipes* (**a**) and *Pleurotus ostreatus* (**b**) treatment groups.

Compared with wheat bran-treated control group, 786 DEGs were identified in *F. velutipes* treatment group, among which 501 genes were upregulated and 285 genes were downregulated (Fig. 6a). Furthermore, 254 DEGs were identified in *P. ostreatus* treatment group, among which 211 genes were upregulated and 43 genes were downregulated (Fig. 6b). The main DEGs related to nutrient composition and energy metabolism are listed in Table 3. As shown in the table, the FPKM value of genes related to the intestinal amino sugar and nucleotide sugar metabolism of BSFL in *F. velutipes* treatment group was 1.57, which was 102.87 times higher than that in wheat bran-treated control group; moreover, the FPKM value of these genes in *P. ostreatus* treatment group was 64.53, which was 41.10 times higher than that in wheat bran-treated control group. In addition, the FPKM value of genes related to the intestinal methionine and cysteine metabolism of BSFL in wheat bran-treated control group was 0.34, which was 44.24 times higher than that in *F. velutipes* treatment group. The FPKM value of these genes in *P. ostreatus* treatment group was 13.88, which was 40.82 times higher than that in wheat bran-treated control group.

**Table 3:** Main differentially expressed genes in various treatment groups.

Gene ID	*Flammulina velutipes* (FPKM)	*Pleurotus ostreatus* (FPKM)	Wheat bran (FPKM)	log_2_ (FC)	*p*. adjust	Functional annotation
gene-LOC119655099	25.62	9.90	1.36	2.6504	0.0003	Cholesterol metabolism
gene-LOC119653395	49.31	49.31	49.31	− 1.2360	0.0000	Valine, leucine and isoleucine degradation
gene-LOC11964t5976	222.75	113.88	131.75	4.4959	0.0308	Phenylalanine metabolism
gene-LOC1196465S5	15.04	13.88	0.34	3.9351	0.0025	Cysteine and methionine metabolism
gene-LOC119658906	70.28	42.04	8.26	2.7278	0.0029	Tryptophan metabolism
gene-LOC11964921S	9.80	25.88	63.90	− 4.0978	0.0178	Alanine, aspartate and glutamate metabolism
gene-LOC119653433	224.33	97.54	45.32	1.8863	0.0049	Pyruvate metabolism
gene-LOC119653555	71.02	54.41	95.71	− 7.0365	0.0001	Starch and sucrose metabolism
gene-LOC11965SS69	30.20	19.36	14.36	9.8759	0.0000	Fructose and mannose metabolism
gene-LQC119646976	222.76	113.88	131.75	4.4959	0.0308	Glycolysis/Gluconeogenesis
gene-LOC119647572	161.50	64.53	1.57	7.5626	0.0000	Amino sugar and nucleotide sugar metabolism
gene-LOC119654254	25.13	26.60	29.81	− 1.1701	0.0011	Galactose metabolism
gene-LOC11964S654	29.33	21.28	14.86	− 1.1083	0.0038	Citrate cycle (TCA cycle)
gene-LOC119654073	8.02	3 55	2.30	− 9.0407	0.0090	Propanoate metabolism
gene-LOC119660564	7.69	5.12	7.46	− 1.8197	0.0216	Glycerophospholipid metabolism
gene-LOC11965310S	2763 03	460.41	56.31	4.6950	0.0212	Biosynthesis of unsaturated fatty acids
gene-LOC119652221	*6.92*	1.40	0.22	4.2972	0.0026	Steroid biosynthesis
gene-LOC119658906	70.28	42.04	8.26	1.7108	0.0018	Glycerolipid metabolism

### GO and KEGG enrichment analyses of DEGs

**Figure 7:**
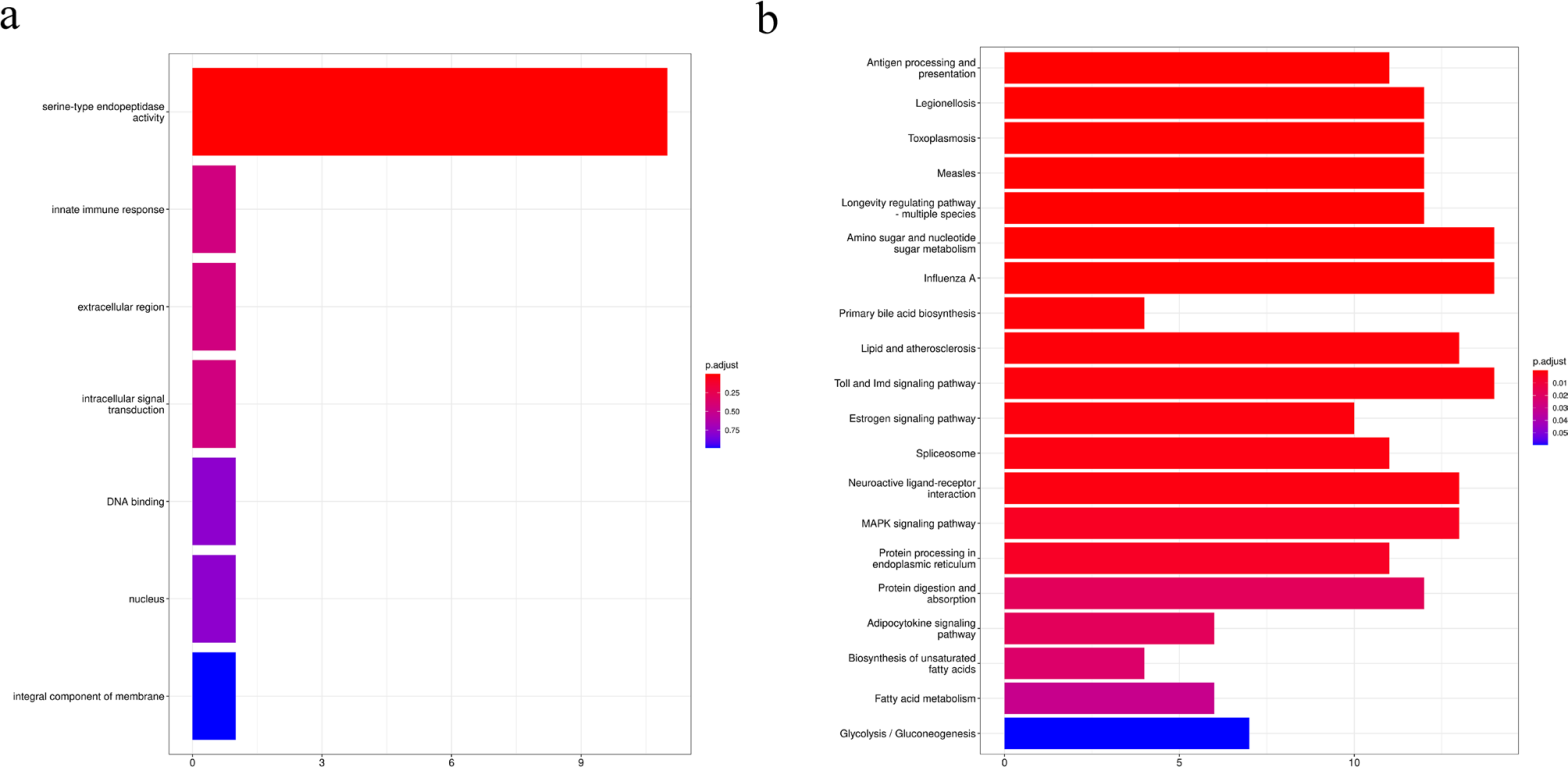
GO (**a**) and KEGG (**b**) enrichment analyses of differently expressed genes between *Flammulina velutipes* treatment group and control group.

Compared with wheat bran-treated control group, GO enrichment analysis of *F. velutipes* treatment group showed that 11 DEGs were enriched in serine-type endopeptidase activity (Fig. 7a). KEGG enrichment analysis revealed that 204 DEGs were enriched in 20 KEGG pathways (Fig. 7b), among which significant enrichment (q < 0.05) was observed in antigen processing and presentation, legionellosis, amino sugar and nucleotide sugar metabolism, and protein digestion and absorption. The category exhibiting the greatest number of enriched DEGs was amino sugar and nucleotide sugar metabolism (14 DEGs), followed by protein digestion and absorption (12 DEGs).

**Figure 8:**
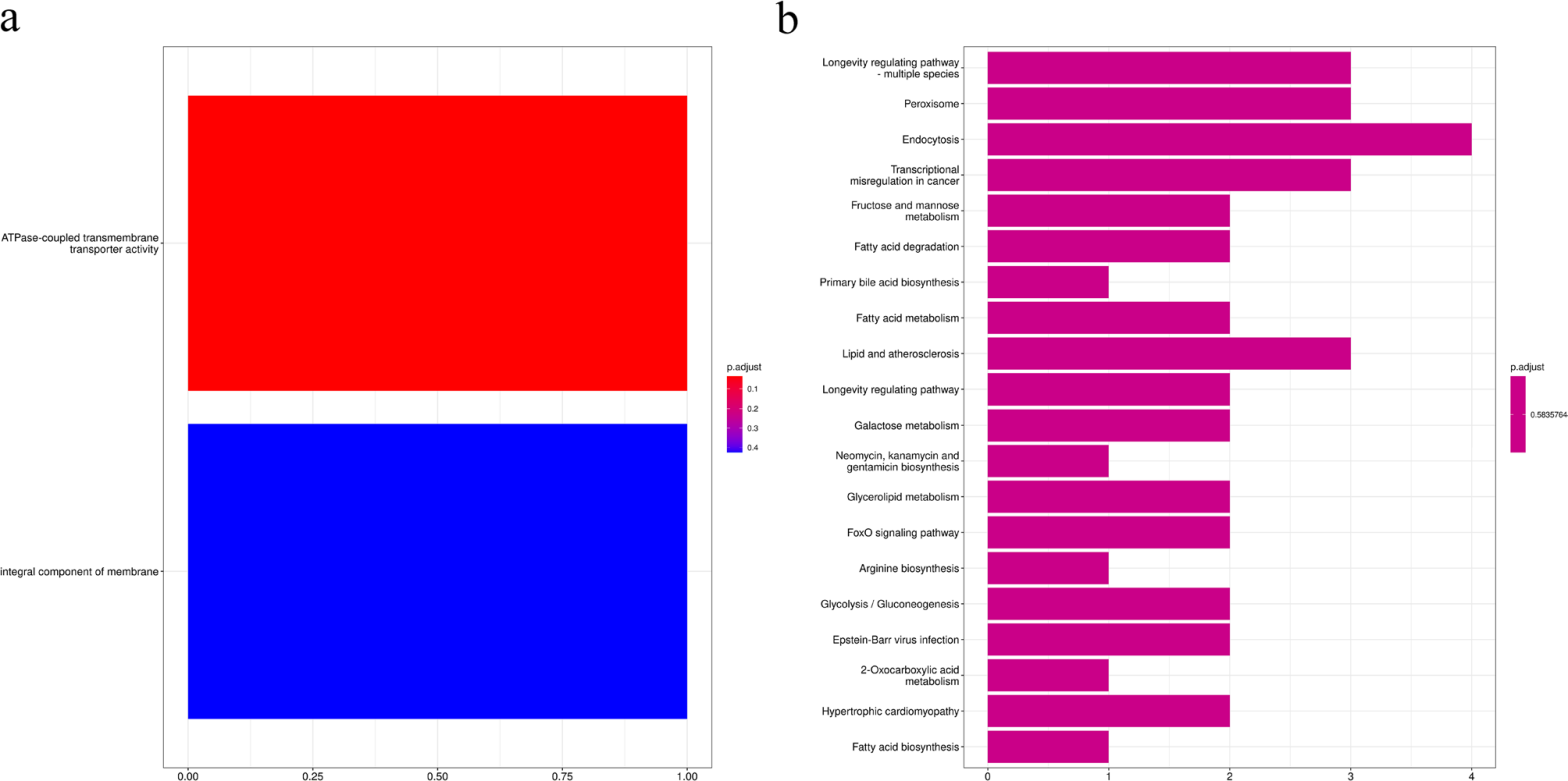
GO (**a**) and KEGG (**b**) enrichment analyses of differently expressed genes between *Pleurotus ostreatus* treatment group and control group.

GO enrichment analysis of *P. ostreatus* treatment group showed that one DEG was associated with ATPase-coupled transmembrane transporter activity (Fig. 8a). KEGG enrichment analysis revealed that 41 DEGs were enriched in 20 KEGG pathways (Fig. 8b), such as fructose and mannose metabolism, fatty acid metabolism, galactose metabolism, and glycolysis/gluconeogenesis; however, these differences were not significant.

## Discussion

In this study, we selected the two most common edible mushrooms in the market as the research subjects for treatment of edible mushroom waste with BSFL. *P. ostreatus*, which has a short growth cycle, is an edible mushroom that is relatively easy to digest and absorb^35^. The cultivation of *F. velutipes* produces a large amount of waste; moreover, it is difficult for mammals to completely digest and absorb the waste because it is rich in dietary fiber. Several studies have attempted to use the waste as an additive in poultry feed^36,37^. In the current study, from the perspective of the remaining amount of feed in different treatment groups, *P. ostreatus* mushroom waste were the most suitable for BSFL feeding, yielding the smallest remaining amount, which was 0.81 times higher than that in wheat bran-treated control group. Second, the largest remaining amount was observed for *F. velutipes* mushroom waste, which was 1.31 times higher than that in wheat bran-treated control group. These results demonstrated that the amount of *F. velutipes* waste remaining after BSFL treatment was greater than the consumption of *F. velutipes* in the natural environment. Based on the difficulty of digesting and absorbing chitin, which is observed in *F. velutipes*, it is speculated that the *F. velutipes* waste are less suitable for BSFL feeding and more difficult to digest among the three types of feed used in this study. This result validates the feasibility of treating edible mushroom foot waste with BSFL. In particular, the amount of *P. ostreatus* waste remaining after BSFL treatment was considerably lower than that remaining after wheat bran control treatment, demonstrating that BSFL can degrade edible mushroom waste.

The relative abundance of intestinal bacterial species in BSFL from different treatment groups varied. Compared with wheat bran-treated control group, the abundance of the cellulolytic bacterial genus *Providencia*^38^ in *F. velutipes* and *P. ostreatus* treatment groups increased by 16.68% and 12.36%, respectively, which were 6.05 times and 4.75 times higher than that of wheat bran-treated control group, with significant differences (*P* < 0.05). *F. velutipes* has a high dietary fiber content and is difficult to digest and absorb. *F. velutipes* and *P. ostreatus* both contain adequate amounts of high-quality protein and are good sources of dietary fiber, vitamin C, B vitamins and minerals^39^. The wheat bran as the control group contained about 70% carbohydrate, 33% pentosan, 8% protein, 2% fiber, and 8.3% fat^40^. The highest crude fat content of BSFL was 28.4%, and the lowest crude protein content was 38%^41^. To meet their growth requirements, BSFL change the abundance of *Providencia* individuals in their gut, to enhance the decomposition and use of dietary fiber. Compared with wheat bran-treated control group (1.42%), the abundance of *Pseudochrobactrum*^42^ in *F. velutipes* treatment group (3.07%) and *P. ostreatus* treatment group (4.33%) increased by 1.65% and 2.91%, respectively, which were 2.16 and 3.05 times higher than that of the wheat bran-treated control group, with significant differences (*P* < 0.05). *Morganella* is a conditional pathogenic bacterium of the gut, and *F. velutipes* can exert immune regulatory effects by stimulating immune responses, producing cytokines, and exerting antibacterial, antiviral, and antifungal effects^43,44^, resulting in lower levels of this bacterium in the gut of the BSFL-treated groups. Studies have also shown that changes in insect diet can affect their immune system while affecting their growth^45,46^. After feeding BSFL with agricultural waste and liquid waste, it was observed that their antibacterial peptides were significantly increased^47^. Subsequently, strains with cellulase activity, protease activity, urease activity, and asparaginase activity were isolated from the gut of BSFL, among which bacteria in the *Providencia* genus were identified as strains with significantly increased abundance after feeding the larvae with edible mushroom waste, thus further verifying the degradation ability of BSFL on edible mushroom waste. In a subsequent, unpublished study, we successfully screened protease-producing strains from the BSFL intestine and demonstrated that they can enhance BSFL intestinal protease activity. In summary, the gut flora of BSFL can regulate the abundance of different bacterial species based on the nutrient composition of the edible mushroom waste. With the assistance of the gut flora, BSFL can better degrade edible mushroom waste and convert them into their own biomass, thus reducing environmental waste while becoming a more nutritious feed source.

The FPKM value of genes related to the intestinal amino sugar and nucleotide sugar metabolism of BSFL in *F. velutipes* treatment group was 161.50, which was 102.87 times higher than that in wheat bran-treated control group (1.57). Furthermore, the FPKM value of these genes in *P. ostreatus* treatment group was 64.53, which was 41.10 times higher than that in wheat bran-treated control group. In addition, the FPKM value of genes related to the intestinal methionine and cysteine metabolism of BSFL in *F. velutipes* treatment group was 15.04, which was 44.24 times higher than that in wheat bran-treated control group (0.34). The FPKM value of these genes in *P. ostreatus* treatment group was 13.88, which was 40.82 times higher than that in wheat bran-treated control group. Feeding with mushroom waste upregulated the expression of intestinal energy metabolism-related genes of BSFL, thus enhancing the absorption and metabolism of energy substances. From the perspective of metabolic pathways, KEGG analysis revealed that compared with wheat bran-treated control group, the intestinal DEGs of BSFL in *F. velutipes* treatment group exhibited significant enrichment in energy-related metabolic pathways, such as amino sugar and nucleotide sugar metabolism and protein digestion and absorption (*P* < 0.02). Taken together with the changes in the intestinal microbial community of BSFL, these findings indicated that because of the difficulty in digestion and utilization of dietary fiber and polysaccharides present in mushroom waste by the black soldier fly, BSFL are forced to upregulate the expression of energy metabolism-related genes. This increases the abundance of bacteria with intestinal digestive enzyme activity, thereby enhancing the absorption and utilization of nutrients.

In summary, this study used BSFL as a treatment medium to organically degrade mushroom waste, which are a waste product of the edible mushroom industry. Mushroom waste showed a certain impact on the abundance of different types of bacteria in the gut of BSFL and expression of gut genes, ultimately increasing the consumption of mushroom waste by BSFL. In conclusion, BSFL can be used to treat organic mushroom waste, thus providing a new alternative for treating edible mushroom waste and protecting the highland environment.

### Relevant legislations, permitting and consent

All experimental procedures were performed in accordance with the standards of the Animal Management Committee of Guizhou Medical University, China.

## Data Availability

The data that support the findings of this study will be available in at https://www.ncbi.nlm.nih.gov/, the 16srDNA sequence of the microorganisms with accession number PRJNA1058849; the gut transcriptome sequencing data with accession number PRJNA1062440.
